# Supplementary figures and images for: Stimulation of the human mitochondrial transporter ABCB10 by zinc-mesoporphrin
Source: PLoS One. 2020 Nov 30;15(11):e0238754. doi: 10.1371/journal.pone.0238754 (PMC7703921; doi:10.1371/journal.pone.0238754)

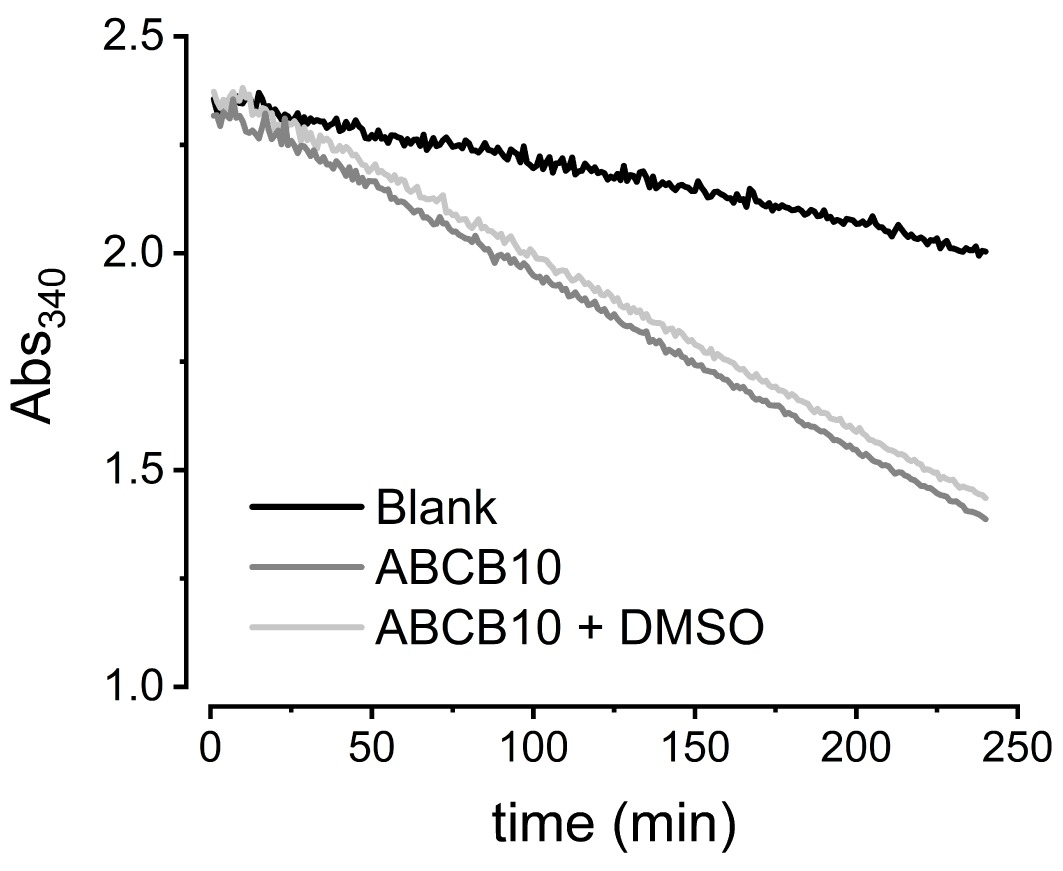

Supplement: S1 Fig — The slope of the absorbance (340 nm) decay depends on the ATP hydrolysis rate of the transporter. The blank represents a control without ABCB10 (black curves). (A) The activity of ABCB10 was not affected by addition of DMSO (2% final concentration), as it is evident from the parallel lines of ABCB10 samples with or without DMSO. This equivalent concentration of DMSO was present in the samples after porphyrins dissolved in DMSO were added to the assay mix. (TIF) [file pone.0238754.s001.tif]

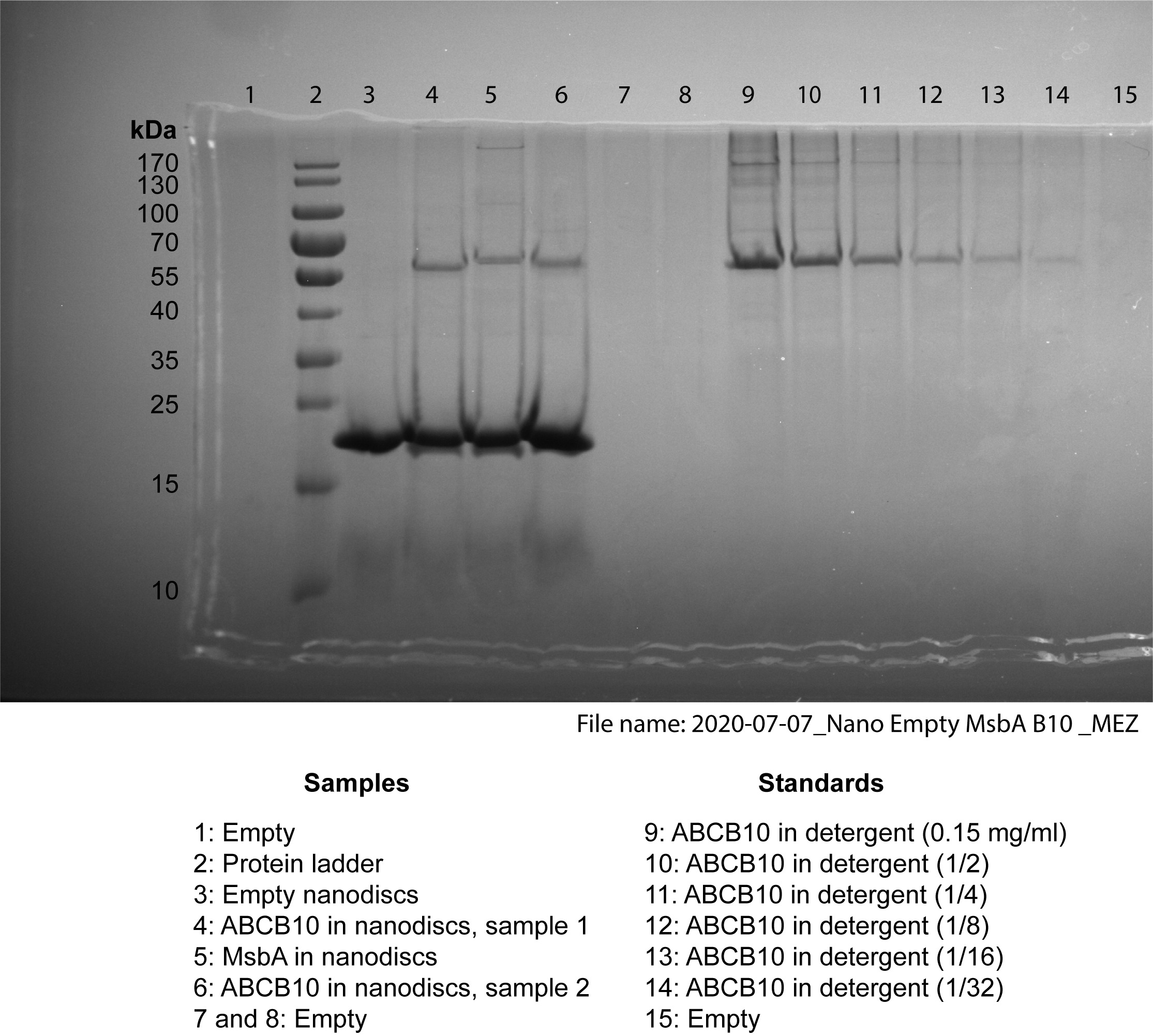

Supplement: S2 Fig — Pre-stained protein ladder (6 μl, Thermo Scientific PI26616) or 20 μl of sample (15 μl protein + 5 μl 4X loading buffer) were loaded per well. The 10% SDS gel was run for 45 minutes at 150V and then stained with Coomassie brilliant blue. Information about the lanes is indicated in the figure. Molecular weight of the ladder bands is indicated at the left (in kDa). (TIF) [file pone.0238754.s002.tif]
